# Novel Processes for the Production of Continuous Carbon Fiber-Reinforced Thermoplastic Polymers via Additive Manufacturing and Comparisons

**DOI:** 10.3390/polym17050584

**Published:** 2025-02-22

**Authors:** Simon Zeidler, Nikolas Matkovic, Florian Kößler, Alexander Puchta, Jürgen Fleischer

**Affiliations:** wbk Institute of Production Science, Faculty of Mechanical Engineering, Karlsruhe Institute of Technology (KIT), Kaiserstaße 12, 76131 Karlsruhe, Germany

**Keywords:** additive manufacturing, laser sintering (LS), Arburg Polymer Freeforming (APF), fused filament fabrication, continuous fiber-reinforced polymer (CoFRP) parts

## Abstract

Continuous fiber-reinforced polymer (CoFRP) parts offer significant potential for reducing future product consumption and CO_2_ emissions due to their high tensile properties and low density. Additive manufacturing enables the tool-free production of complex geometries with optimal material utilization, making it a promising approach for creating load-path-optimized CoFRP parts. Recent advancements have integrated continuous fibers into laser sintering processes, allowing for the support-free production of complex parts with improved material properties. However, additive manufacturing faces challenges such as long production times, small component dimensions, and defects like high void content. New processes, including Arburg Polymer Freeforming (APF), robotic direct extrusion (DES) and the integration of thermoplastic tapes, and laser sintering, have enabled the production of CoFRPs to address these issues. A comparison of these new processes with existing material extrusion methods is necessary to determine the most suitable approach for specific tasks. The fulfillment factor is used to compare composites with different matrix and fiber materials, representing the percentage of experimentally achieved material properties relative to the theoretical maximum according to the Voigt model. The fulfillment factor varies significantly across different processes and materials. For FFF processes, the fulfillment factor ranges from 20% to 77% for stiffness and 14% to 84% for strength, with an average of 52% and 37%, respectively. APF shows a high fulfillment factor for stiffness (94%) but is lower for strength (23%), attributed to poor fiber–matrix bonding and process-induced pores. The new DES process improves the fulfillment factor due to additional consolidation steps, achieving above-average values for strength (67%). The CoFRP produced by the novel LS process also shows a high fulfillment factor for stiffness (85%) and an average fulfillment factor for strength (39%), influenced by suboptimal process parameters and defects.

## 1. Introduction

Continuous fiber-reinforced polymer (CoFRP) parts possess immense potential for effectively and economically reducing future product consumption and CO_2_ emissions [1]. This is due to the high mechanical tensile properties along the fiber direction paired with the low density of the CoFRP parts, which leads to a reduction in the component’s weight [2]. Additive manufacturing processes allow for the tool-free production of complex geometries with optimal material utilization. In combination, this offers a promising approach for the production of load-path-optimized CoFRP parts without the need for tools, allowing for efficient production with high customization and complex shapes. Acknowledging this potential, for some additive processes, continuous fiber (co-fiber) reinforcement has been developed. The largest area of focus up until now has been the field of material extrusion (MEX) processes [3]. The nature of these processes necessitates the use of support structures, which must be removed and disposed of after production. This ultimately results in additional time and costs for disposal and post-processing steps. Further, the use of support structures restricts the possibility of overhangs, cavities, and undercuts, thus limiting the complexity of the parts. The removal of support structures also impacts the surface quality. The latest developments observed for MEX are multiaxial and non-planar 3D printing. With multiaxial 3D printing, additional rotational axes are used in addition to the three linear axes in the x, y, and z directions [4]. This allows for the orientation of the nozzle to be changed during printing, which opens up better surfaces and new possibilities, for example. With non-planar 3D printing, in contrast to conventional MEX printing, the z height can vary during the printing of a layer [5]. This can be achieved with the conventional three axes but can also be combined with multiaxial 3D printing. As conventional industrial robots have six axes, they are often used for multiaxial and non-planar printing. In addition to the MEX processes, there are further efforts to integrate fibers into other additive processes. Only recent research has shown the possibility of integrating continuous fibers into the laser sintering process [6]. Compared to the MEX process, laser sintering offers the advantage that no support structures are required, as the powder bed supports the model. This makes more complex geometries possible. Laser sintering also ensures more stable, isotropic components with greater attention to detail and a smoother surface. It enables a greater variety of materials and more efficient production, as several parts can be manufactured at the same time [7]. However, the costs are usually higher than with MEX. Given all the advantages, the combination of fiber integration and laser sintering is very promising. Extensive studies of the developed process have already been carried out [6,8,9,10,11,12].

The disadvantages of most additive approaches are the long production times and small component dimensions, with a generally low output rate and various defects such as a high void content in the produced composite [13,14]. Hence, another new process for the production of CoFRP parts with a focus on the production of larger-scale parts was developed in the meantime. This, as of now, is mostly realized with the help of industrial robots, with a focus on discontinuous fiber reinforcement. The integration of continuous fibers is not currently feasible regarding economic production. Therefore, a newly developed process addresses this challenge through the use of thermoplastic tapes added onto the printed contour [15]. By using six-axis industrial robots, this new process also investigates the combination of fiber integration with the possibilities of multiaxial and non-planar 3D printing. Apart from in very isolated state-of-the-art approaches, this has not yet been investigated much, but its potential has been shown [16].

Newly developed processes need to be compared to existing and established processes to allow for the selection of a suitable process for a given task. Therefore, research on the production of continuous fiber-reinforced parts with MEX will be compared with three newly developed processes for continuous fiber reinforcement, namely, Arburg Polymer Freeforming (APF), laser sintering (LS), and robotic direct extrusion (DES). Before the comparison, the principles of the different processes will be briefly explained.

## 2. CoFRP in Material Extrusion Processes

Reinforcement fibers in additive manufacturing are most widely used with MEX processes. These processes use a solid feedstock, which is melted and extruded through a heated nozzle. The most popular among these processes is fused filament fabrication (FFF).

### 2.1. Fused Filament Fabrication

In the context of fused filament fabrication (FFF), the feedstock takes the form of a polymer wire, known as filament. The process commences with a spool of filament, which is fed into a heated extruder. The extruder melts the filament and deposits it onto a build platform, following a predetermined path that is computer-controlled. As the material cools and solidifies, it forms a solid layer. The build platform then moves down slightly, and the next layer is deposited on top of the previous one. This process is repeated until the entire object is complete. One of the main advantages of FFF is its accessibility and affordability. It is widely used by hobbyists, educators, and professionals due to the relatively low cost of the printers and materials. The technology supports a variety of thermoplastic materials, including PLA, ABS, PETG, and TPU, each offering different properties [17]. Despite its benefits, FFF has some limitations. The layer-by-layer construction can result in visible layer lines and a rough surface finish, which may require post-processing to achieve a smoother appearance. Furthermore, the mechanical properties of FFF-printed parts can be anisotropic, meaning they vary depending on the direction of the layers, which can affect the strength and durability of the final product [18].

#### 2.1.1. Integration of Continuous Fibers with FFF

**Figure 1:**
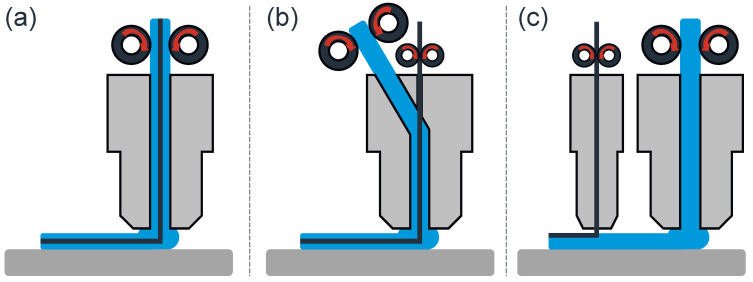
Different methods of co-fiber (black) integration in fused filament fabrication (polymer depicted in blue): (**a**) extrusion of pre-impregnated continuous fiber filament, (**b**) separate feeding of fiber and filament with in situ impregnation, (**c**) dual-nozzle extrusion with separate nozzles for fiber and filament [15,20].

In order to integrate continuous carbon fibers into the FFF process, three main principles are employed (see Figure 1). Firstly, the extrusion of pre-impregnated continuous fiber filaments is possible. Secondly, the extrusion with separate feeding of the fiber and filament and in situ impregnation is used. Lastly, dual-nozzle extrusion is applied [19].

The utilization of pre-impregnated fibers confers several benefits when contrasted with the alternative two processes. Primarily, these benefits comprise the eradication of voids that are typically formed during impregnation with a highly viscous thermoplastic. Another advantage is the increase in bonding strength between the fiber and the matrix, resulting from adequate consolidation during the production of the pre-impregnated fiber [21]. However, continuous reinforced filaments are not widely available. For extrusion and in situ impregnation [19], three methods were identified. First, a common entrance for the fiber and filament into the extruder can be used (Figure 1a). However, this method is susceptible to errors arising from the inability to adjust the feeding rates, which can result in filament curling and subsequent fiber damage. To address this limitation, a second method has been developed that utilizes distinct entrances (Figure 1c). The third method involves a central feeding of the fiber with the impregnation of molten filament from another direction (Figure 1b), which offers a solution for aligning the fibers centrally. However, it should be noted that the fiber matrix bonding is inferior to that achieved with pre-impregnated fibers. The third method involves dual-nozzle extrusion, where fiber and thermoplastic are consolidated within the part itself. In this process, the fiber is extruded into the extruded filament, which remains in a molten state [19]. The alignment of the carbon fibers can be regulated through the printing process for all three methods, enabling the optimization of the part’s strength in specific orientations.

#### 2.1.2. Process Parameters Affecting the Produced Parts

In the FFF process, a multitude of parameters influence the properties of the resultant component. For polymers, the properties typically increase with an increase in time, temperature, and pressure [22]. In the context of FFF, ref. [23] enumerated a range of parameters, including layer thickness, build orientation, raster angle/orientation and width, air gap, extrusion temperature, print speed, infill pattern and density, nozzle diameter, and the number, width, and the air gap of the contours. These parameters are designed to enhance the impregnation of fibers with the matrix material, which plays an important role in facilitating load transfer into the fibers. As impregnation levels rise, the capacity for load introduction into the fibers is augmented due to an enhancement in the fiber–matrix interface. This, in turn, leads to an increased reinforcement effect of the fibers [24]. Conversely, void formation along the fiber mitigates the reinforcement effect. Voids may arise from the quality of the raw materials or from gas expansion during the melting of the thermoplastic [25]. In both cases, pressure is a process parameter that affects impregnation and void formation. Secondly, refs. [26,27] traced void formation back to poorly controlled deposition processes, including insufficient flow of the matrix material, unmatching layer heights and nozzle diameters, and poorly or not optimized tool paths. Refs. [28,29,30] concluded that void reduction can be achieved by adjusting the layer thickness.

#### 2.1.3. Properties of CoFRP by FFF and Evaluation

**Figure 2:**
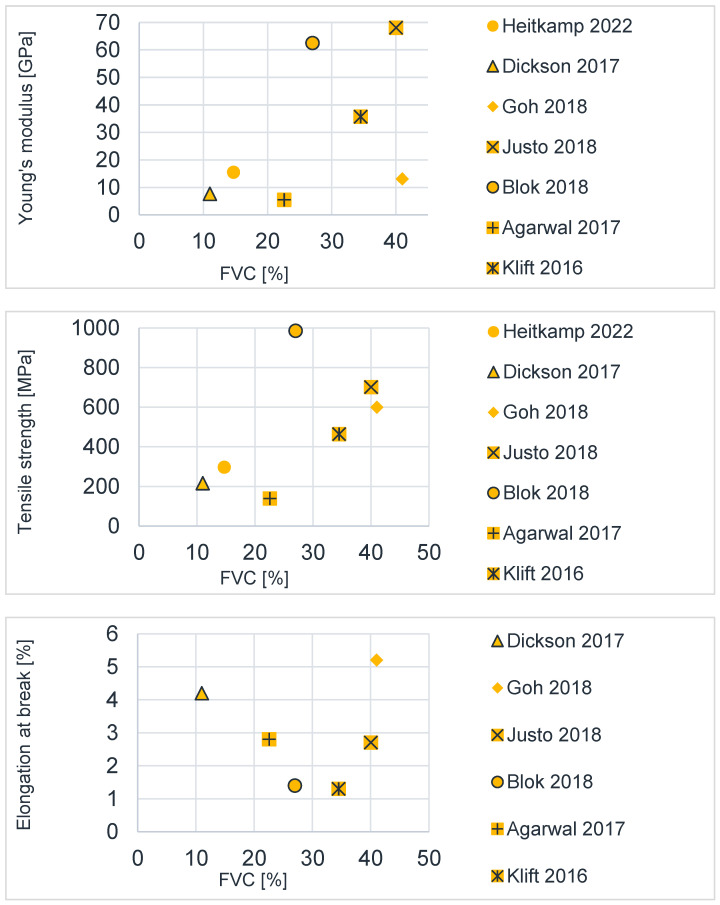
Mechanical properties of FFF-produced CoFRP [13,33,34,35,36,37,38] in [6].

The Young’s modulus of CoFRP by FFF is contingent upon the fiber volume content (FVC) of the produced part. Figure 2 shows the values of the achieved tensile strength, Young’s modulus, and elongation at break for selected CoFRP produced via FFF. As can be seen, the mechanical properties increase almost linearly with increasing FVC, for which typical values range between 10% and 40% for additively produced parts. The observed variations in mechanical properties can be attributed to the utilization of distinct matrix or fiber materials, as well as variations in the quality of bonding between the fibers and the matrix. The pronounced anisotropy observed in FFF is primarily attributed to the inadequate adhesion of the polymer strands. During the extrusion process, a temperature gradient inevitably arises between adjacent strands, resulting in residual stresses and reduced adhesion of adjacent melt strands. In addition to the temperature gradient, geometric notches and, thus, defects are continually introduced into the component, which further reduces the component strength [31]. Consequently, FFF is primarily employed for prototypes [32].

### 2.2. Arburg Polymer Freeforming

The Arburg Polymer Freeforming (APF) process is an additive manufacturing process that shares similarities with the FFF process. However, it is based on the injection molding process, which means it utilizes commercial thermoplastic granulates that are typically processed in the injection molding process. In contrast to FFF, the polymer for the additive manufacturing of the component is not extruded in strands for APF but, rather, due to a timed nozzle shutter, is dispensed drop by drop through a needle valve [39]. The APF process control is based on the drop characteristic number, also known as the discharge number, which exhibits linear proportionality with the volume of each individual drop and represents the controlled variable. The mean feed rate of the screw in the injection molding cylinder is calculated based on a large number of drops and the diameter of the cylinder, thus determining an average value for the volume of the individual drops. The control variable is the pressure in the injection molding cylinder, which forces the molten material out of the nozzle opening at different speeds depending on its viscosity, which in turn depends on the material and temperature.

#### 2.2.1. Integration of Continuous Fibers with APF

**Figure 3:**
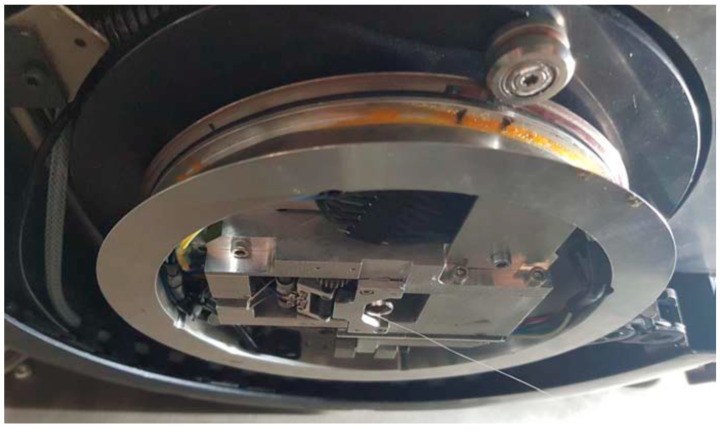
Fiber Integration Unit in Arburg Polymer Freeforming [39].

The system technology’s initial generation utilizes a module comprising a unit that rotates concentrically around the plastic discharge, thereby facilitating the conveyance of a fiber yarn (see Figure 3). The fibers conveyed under the plastic discharge are directly overprinted by the discharged plastic, thus becoming integrated into the component. The module’s primary functions include the alignment of the fibers and their subsequent conveyance. The separation of the fibers is achieved through a process of cutting with a blade at the edge of the discharging cannula [39]. In the subsequent generation, a fixed extrusion was employed for the fiber, while a movable and turnable table was utilized for the tool path. The implementation of reinforcing fibers is straightforward, as only the initiation of the fiber feed is required. With the appropriate orientation and dispensing speed, the fibers are automatically positioned correctly and can be integrated into the component by the plastic discharged through the Freeformer nozzle. The method for separating the fibers is more complex. To do so, the fiber roving, which is partially imprinted in the component, must be moved against its actual discharge direction so that it is bent around the edge of the cannula, broken in the process, and, thus, successfully separated. The efficacy of this separation method hinges on the stability of the fiber’s position within the discharge unit and the component. The presence of rubberized feed rollers serves to prevent slippage, yet the possibility of fiber peeling off the component remains if the force acts in a direction counter to the implementation. To address this, the fiber implementation module is rotated 180°, and the build platform is moved a predetermined distance in the direction of fiber discharge set before the rotation. This ensures that no peel forces act on the implemented fiber. The polymer used in this research is ABS Terluran GP35 (INEOS Styrolution Group GmbH, Frankfurt am Main, Germany), chosen for its technical relevance. As fiber material, an E glass fiber yarn (type EC9-34Z28 34 tex) (FULLTECH FIBER GLASS CORP., Taipei, Taiwan) is used. For better processability, it is coated with PERICOAT AC 250 (Textilchemie Dr. Petry GmbH, Reutlingen, Germany) at the DITF Denkendorf; the additional application is 12.5% based on the fiber weight.

#### 2.2.2. Process Parameters Affecting the Produced Parts

Baumann’s investigation focused on the process–property relationships of the developed APF with fiber integration. The parameters adjustable at the Freeformer are listed below. The droplet index, which indicates the volume per plastic droplet, must be adapted to the desired production resolution. Varying the droplet index alone will inevitably lead to overfilling or underfilling of the component and is, therefore, not advisable, even if a certain effect on the mechanical properties could be achieved. The pressure difference is the primary control variable, governing the adjustment of the drop characteristic number for the freeform. It quantifies the extent to which the pressure must be altered to achieve the desired drop characteristic number. It has been established that a greater pressure difference results in a more rapid adjustment of the drop characteristic number to the target value, though this also leads to increased fluctuations around the target value. The value exerts a significant influence on the consistency of the component filling in both reinforced and unreinforced areas. Given its role in regulating the aforementioned drop size distribution, a low dependency between the pressure difference and the mechanical properties of the FRP samples is anticipated. The temperature profile of the cylinder pertains to the temperatures of the various heating zones. The remaining parameters of the dosing unit, including the dosing speed, dosing distance, back pressure, and others, have a general effect on plastic discharge, thereby affecting both reinforced and unreinforced areas. These parameters are utilized to optimize material processing. The preload force of the piezo actuator is defined as the force with which the piezo actuator is braced with the needle diaphragm. It has been established that an increase in this force results in an increase in the closing force of the needle diaphragm. Consequently, a higher melt pressure is required to enable material dispensing. However, this value is not expected to have a significant impact on the mechanical properties of the components, as the melt pressure is increased until the desired droplet index is attained. However, it is important to note that the elevated pressure and the ensuing augmentation in shear forces during the plastic discharge can potentially lead to a slight degradation in the material, which may have a deleterious effect on plastic and fiberglass-reinforced plastic components. It is noteworthy that the impact of these parameters on the properties is minimal. The temperature of the discharge nozzle directly influences the temperature of the material being dispensed. Increasing the temperature of the plastic leads to a reduction in its viscosity, thereby enhancing its capacity to infiltrate and flow around the fibers present. Furthermore, the elevated temperature of the material during discharge can lead to the re-melting of the surface of plastic material that has already solidified, thereby enhancing the bond with the existing plastic. The temperature of the entire manufacturing environment is reflected in the temperature of the build chamber. It has been established that elevated temperatures during the manufacturing process result in reduced cooling rates of the component. This phenomenon leads to a reduction in energy requirements for reheating surfaces that have already undergone cooling. Consequently, an increase in build chamber temperature is expected to enhance the mechanical properties. Additionally, the elevated temperature of discharged fibers contributes to delayed solidification of the plastic, thereby allowing for enhanced infiltration into the fibers and subsequent flow around them. Consequently, an elevated build chamber temperature should yield enhanced mechanical properties for both unreinforced and reinforced components. The fiber integration parameters, namely conveying speed and positional accuracy, were discussed. The conveying speed of the reinforcing fibers should be synchronized with the production speed. If the conveying speed is excessively high, the fibers are compressed, resulting in uneven fiber placement. Conversely, if the conveying speed is excessively low, the fibers are laid in a straight orientation, resulting in even tension. However, this can lead to damage to the feed rollers. Theoretically, the conveying speed exerts a significant influence on the mechanical properties of the FRP test specimens. Nevertheless, it is preferable to convey at the optimum speed, that is, synchronously with the production speed. A close examination of the data reveals a substantial impact of the factors’ nozzle temperature and process chamber temperature on Young’s modulus. A similar observation can be made for tensile strength, where nozzle temperature, process chamber temperature, and FVC prove to be significant factors. Furthermore, the interrelationships between nozzle and process chamber temperature, as well as process chamber temperature and FVC, are found to be noteworthy. In contrast, the interrelationships of nozzle temperature, process chamber temperature, and FVC exhibit either no effect or an insignificant impact on Young’s modulus [39].

#### 2.2.3. Properties of CoFRP by APF and Evaluation

In order to ascertain the processing parameters for manufacturing components with optimal mechanical properties, a parameter study was conducted on unidirectionally reinforced tensile test specimens. To this end, the target variables and influencing factors were first determined, and then a corresponding full factorial experimental design was set up. During the fabrication process, an unreinforced specimen was concurrently produced with each FRP specimen to discern potential correlations resulting from mutual influence or other environmental factors. The detection of these correlations suggests the existence of additional unknown environmental influences and/or non-constant or undefined influencing factors. With a parameter set of 230 °C for the nozzle, 80 °C for the process chamber, and a fiber volume content (FVC) of 21%, a Young’s modulus of 16.2 GPa was achieved. The highest tensile strength was obtained during parameter optimization at an FVC of 9.7% with 186 MPa, employing the same processing parameters. The E-modulus of the APF is notably superior to comparable values reported in the literature for CoFRP, though the tensile strength is typically lower [39]. The fulfillment of the values theoretically possible with the respective FVG is calculated at 95% for the E-modulus and 56% for the tensile strength [39]. The APF presents a promising alternative for the production of CoFRP, given the use of production-grade granulates typically employed in injection molding.

## 3. Robotic Direct Extrusion Systems (DES)

Contrary to conventional FFF printers, MEX direct extrusion systems (DESs) do not necessitate pre-processed filaments. Instead, as the nomenclature implies, they are capable of direct granulate processing. Single-screw extruders, which are mounted on a kinematic system, are frequently employed for this purpose. The printing of components is executed in a manner analogous to FFF, with the melted granulate being applied in layers. In contrast to FFF, DESs exhibit considerably higher output, with discharge rates of 30 kg/h being a common occurrence. DESs typically utilize three-axis gantries or six-axis industrial robots as kinematics, a choice that offers several advantages. These include a smaller system footprint compared to the build volume and the use of the additional three rotational axes for non-planar 3D printing. The absence of filament dependency in DES enables the utilization of a substantially larger number of material systems in the form of granules, which are also considerably more economical than filament-based materials. The employment of short-fiber-reinforced materials is prevalent due to their enhanced thermomechanical properties and comparatively low costs [40,41].

### 3.1. Production of Continuous Fibers with Robotic Extrusion and Tape Laying

**Figure 4:**
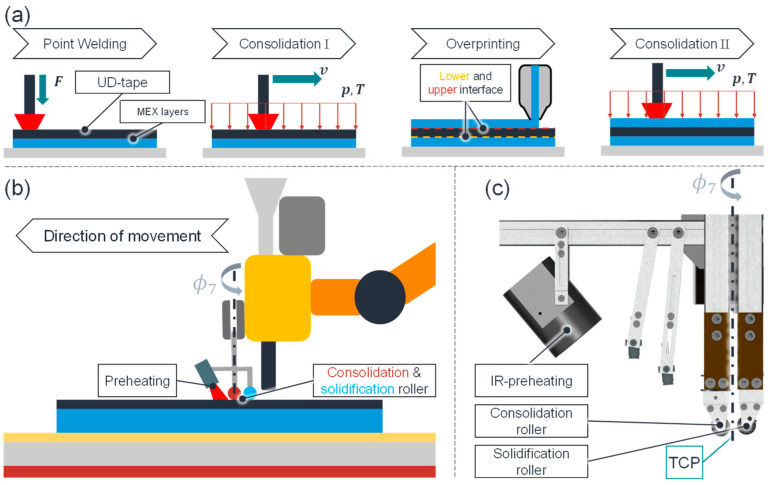
(**a**) Hybridization process; (**b**) concept hybrid end effector for DES and consolidation; (**c**) consolidation tool [15].

In order to circumvent the disadvantages associated with the continuous fiber reinforcement of the two previously mentioned MEX processes, FFF and APF, a novel process was developed, as depicted in Figure 4 [42]. The objective is to produce large-volume CoFRP while evading defects such as voids that can adversely affect fiber–matrix bonding. As illustrated in Figure 4a, the MEX layers are produced using a robotic DES with the aid of targeted consolidation processes (i.e., the application of pressure and temperature) reinforced with unidirectional (UD)-tapes. The UD-tapes are initially secured through a point welding process and subsequently consolidated lengthwise and widthwise in an iterative manner. The fiber volume can be augmented by layering multiple UD-tapes, akin to the tape laying process [43]. To achieve additional MEX layers, the final UD-tape is overprinted and consolidated prior to the printing of subsequent MEX layers. The employment of UD-tapes confers numerous benefits. The introduction of co-fibers can be expedited at a local level, and a high fiber volume content can be achieved (although this is limited by the fiber volume content of the UD-tape). In comparison to rovings in FFF or APF, the fibers exhibit superior impregnation penetration, resulting in more uniform and effective fiber matrix bonding [44].

Given the necessity of alternating between the MEX with the DES and the consolidation process step, a hybrid end effector was developed (see Figure 4b). Thermal simulations have demonstrated that dynamic preheating is essential for consolidation [15]. Consequently, an infrared (IR) heater with a temperature control loop was constructed (see Figure 4c). The consolidation tool comprises a heated roller and a non-heated solidification roller. The presence of the latter is intended to briefly maintain pressure after the actual consolidation and, thereby, avert deconsolidation of the still-warm FRP.

**Figure 5:**
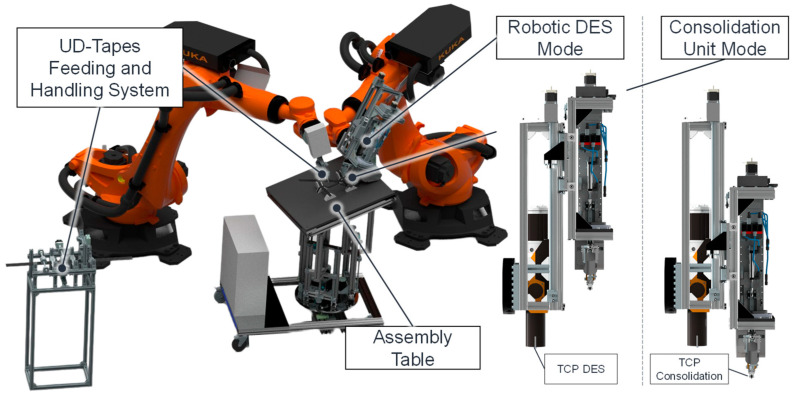
Overview of the robotic system for DES with continuous fiber reinforcement [15].

The robotic system developed for the process is depicted in Figure 5. The system principally consists of two industrial robots with suitable end effectors, a UD-tape feeding unit, and a heated table with parallel kinematics as a workspace. The right robot handles the hybrid end effector, which can quickly switch between DES and consolidation modes. The left robot is equipped with a heated gripper that is used to handle and form the UD-tapes [45]. The UD-tapes, which are provided in the appropriate length by an automatic feeding unit with a cutting mechanism, are then consolidated using a pneumatic pressure control consolidation unit that enables a defined force to be applied during the consolidation process.

### 3.2. Process Parameters Affecting the Produced Parts

In order to gain a more profound understanding of the process, several experimental designs were set up to identify the process parameters with the highest effect. The effect considered here is the maximum achievable tensile shear strength of the FRP between the UD-tape and the MEX layer. For this purpose, samples were produced based on the ASTM D5868-01 standard [46]. The material system used was TenCate Cetex TC910 UD-tapes (Toray Advanced Composites, Morgan Hill, CA, USA) with a PA6 matrix and 43 volume-% carbon fibers. For the MEX layers, PA6 granules with 30 volume-% carbon short fibers were used.

**Figure 6:**
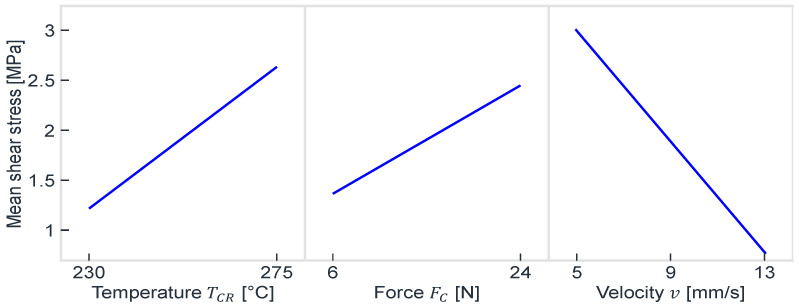
Main effect diagrams of Consolidation I for PA6 with carbon fibers [15].

Figure 6 shows the main effects for the process step Consolidation I (see Figure 4a) for a 2^2^ × 3^1^ experimental design. In each instance, eleven samples were produced for each factor-level combination, ensuring the attainment of substantial results due to the high degree of scatter present. The factor levels encompassed the two consolidation temperatures (TCR), the two consolidation forces (FCR), and the three consolidation velocities (*v*), as illustrated. The specific values for the factor levels, and consequently the process window, were ascertained through preliminary studies. Particular attention was paid to the maximum values for the consolidation temperature and the force to ensure that the composite was not damaged. A velocity of 5 mm/s was selected so as not to make the process unnecessarily long. As can be seen, the consolidation velocity has the greatest absolute effect within the permitted process window, if one looks at the increase in mean shear stress from 13 mm/s to 5 mm/s. A low-consolidation velocity enables the consolidation roller to maintain pressure in an area for an extended period, thereby allowing the MEX layers’ polymer chains and the UD-tapes’ matrix sufficient time to interdiffuse. In principle, a lower consolidation velocity could be selected to enhance the consolidation outcome, provided that the process duration is acceptable. The consolidation force exerts the least influence, as the upper limit of the process window was set relatively low to prevent damage to the composite.

For Consolidation II, the main effects were determined, equivalent to Consolidation I. The analysis revealed that the factors exerted minimal influence on the attainable tensile shear strength of the material system under investigation.

The overprinting process (refer to Figure 4a) was examined employing the same methodological framework as the consolidation process steps. The findings indicated that an over-extrusion of approximately 30% during the overprinting process of the UD-tape resulted in an enhancement of the tensile shear strength by approximately 19%. It was observed that other process parameters, such as the extrusion temperature, exhibited a gradual response and could only be adjusted to a limited extent during the overprinting process. Consequently, these parameters were not further investigated.

### 3.3. Properties of CoFRP by Robotic Extrusion and Tape Laying

To validate the developed process, tensile samples were produced based on the standard DIN EN ISO 20753 [47] for the material system PA6 with the carbon fibers mentioned in the previous section

The tensile tests were repeated 11 times for each specimen, and the tensile strength of the specimens without UD-tape was found to be 61.3 MPa. In contrast, the tensile strength of the specimens with a layer of UD-tape along the center of the tensile specimen was found to be 151.5 MPa, representing an increase of 147%. Despite comprising merely 1.65% by volume of the tensile specimen, the co-fibers of the UD-tape exhibited a substantial enhancement in tensile strength, underscoring the efficacy of the consolidation process in facilitating robust interfacial bonding between the MEX layers and the UD-tape.

The newly developed process for combining DES and tape laying enables the expeditious production of large-volume thermoplastic components with continuous fiber reinforcement. The UD-tapes are characterized by optimal handling and effective impregnation, which, in conjunction with targeted consolidation, ensures optimal bonding within the component. A notable advantage of the MEX layers is their ability to be constructed from a broader range of thermoplastic material systems, resulting in significantly reduced costs compared to conventional FFF with filament, due to the direct melting of granulate in their fabrication. However, it should be noted that the process is primarily suited for relatively uncomplicated and flat components.

## 4. CoFRP in Laser Sintering

According to ISO 52900 [48] and VDI 3405 [49], (selective) laser sintering (LS) is a powder bed-based process (powder bed fusion, PBF-LB/P) for the additive manufacturing of polymer components that uses a laser to fuse small particles of polymer powder into a solid structure based on a digital 3D model. The process involves spreading a thin layer of polymer powder onto a build platform, which is subsequently subjected to selective melting by a laser in accordance with the design specifications. Upon completion of a layer, the platform is lowered, and a new layer of powder is applied and sintered. This layer-by-layer approach facilitates the fabrication of complex geometries without the necessity for support structures. The utilization of LS in the fabrication of functional prototypes, end-use components, and intricate designs is pervasive in industries such as aerospace, the automotive sector, and healthcare, owing to its capacity to yield robust, durable components with high precision [32].

### 4.1. Laser Sintering of Polymers

**Figure 7:**
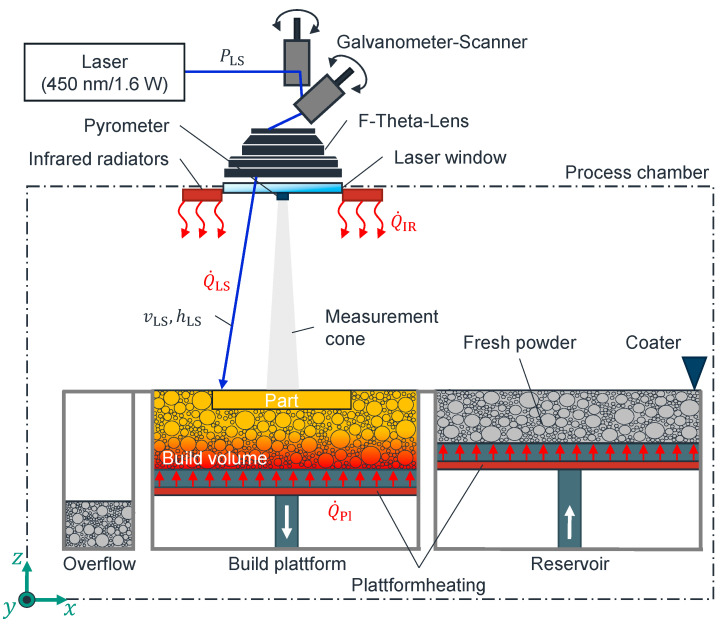
Process schematic of laser sintering [6].

The core components of an LS machine are as follows: firstly, a laser for selectively melting the powder; secondly, a galvanometer scanner to deflect the laser beam; thirdly, an optical system for focusing the laser beam on the powder bed surface; and fourthly, a laser window as a transition between the process chamber and the focusing optics. The machine also contains a thermally insulated process chamber with a build platform, a powder supply, and a coater. Roller or blade coaters are used in industry. Infrared radiators (IRs) are placed above the build platform. These IRs are used to achieve the most homogeneous heating possible within the polymer’s process window of the component or powder bed. Additional heat sources on the container walls or platform floors are used to control the temperature of the powder bed and to prevent cool down during the process (see Figure 7). The resulting properties of LS components are significantly influenced by the temperature control in the process chamber and powder area, as well as by the energy input of the laser beam [32].

### 4.2. Machine and Process for Integration of Continuous Carbon Fiber Rovings

**Figure 8:**
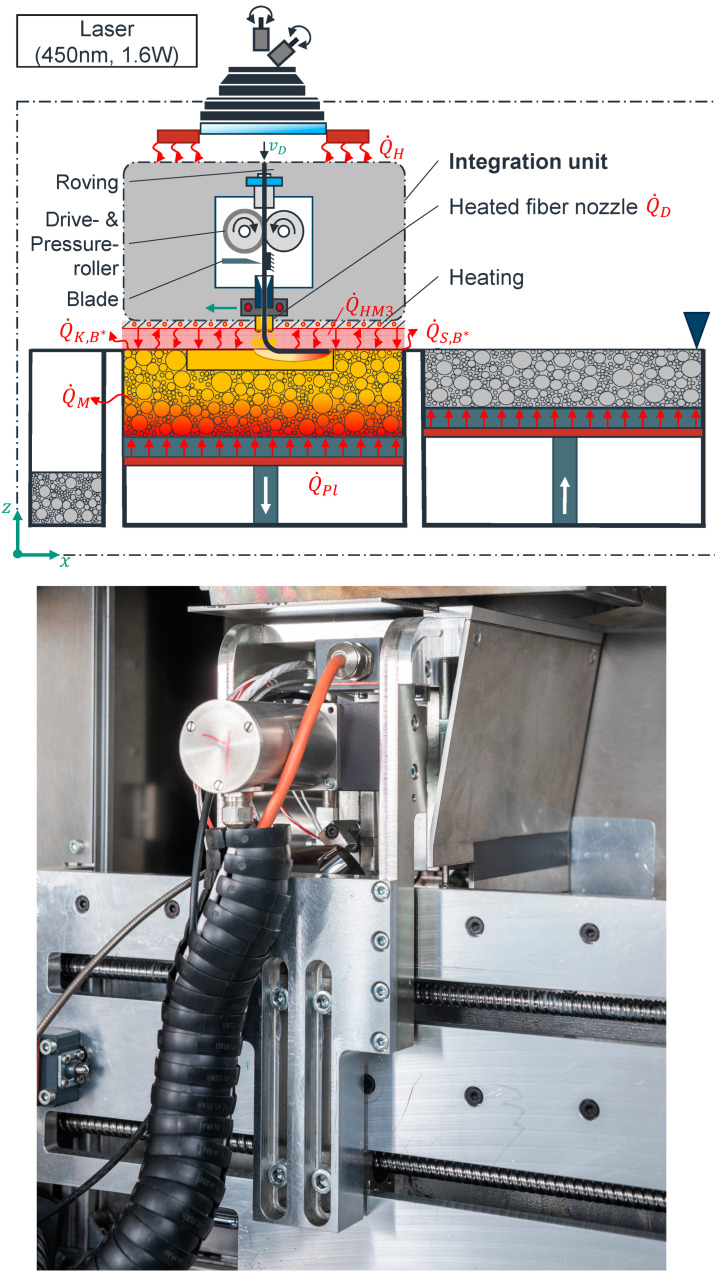
Process schematic (**top**, [6]) and fiber integration unit (**bottom**, ©KIT, Amadeus Bramsiepe) of the developed process.

Figure 8 shows the developed LS machine. In order to enable the layer-by-layer construction process of components in the developed LS machine, a galvanometer scanner, a diode laser, a blade coater, an F-Theta lens, a direction (z), a construction, and a powder storage container that can be moved in the construction direction (z), as well as various heat sources (IR emitters (Excelitas Noblelight GmbH, Kleinostheim, Germany), platform heating (Seibert Vertriebs GmbH, Mühlacker, Germany)) with temperature control (pyrometer (LumaSense Technologies GmbH, Frankfurt am Main, Germany), PT100 (otom Group GmbH, Bräunlingen, Germany)) form the core elements of the LS machine. A fiber integration unit installed in the process chamber is used for layer-specific integration of rovings. According to Figure 8, this can be moved two-dimensionally along the *X* and *Y* axes [6].

As illustrated in Figure 8, the fiber integration unit comprises a roving extruder, equipped with a step motor and a drive roller that facilitates the downward feeding of the roving into the designated direction. It also incorporates a pressure roller, ensuring a slips-free feeding process, and a cutting blade that precisely cuts the roving. Additionally, an auxiliary heat source positioned at the base of the fiber integration unit is utilized to maintain the component and powder bed surface at a specific temperature within the process window. The latter is composed of a black-painted metal sheet and a glued-on silicone heating mat. The fiber nozzle is heated by a heating element [6]. The fiber integration unit moves over the start point of the first roving path for integration of the roving in a user-defined layer. During the entire duration of the roving integration, the IR radiation and the measuring cone of the pyrometer are blocked by the fiber integration unit. Consequently, the supplied heat flow of the IR emitters is rendered ineffective. The built-in diode laser is kept inactive during the roving integration. To prevent the component or powder bed surface from cooling, the component or powder bed surface is maintained at a suitable temperature within the process window by the heat source located on the underside of the fiber integration unit. 

As a result of the heat flow between the heated fiber nozzle and the powder bed surface, a melting zone with a characteristic width and depth is created in the already-fused layers of the component. During the heat exposure, the viscosity level of the polymer is locally reduced, and, thus, the polymer is increasingly liquified.

At the same time, the roving is fed and bent by the mentioned drive and pressure roller synchronously with the feed motion of the fiber integration unit between the front side of the fiber nozzle and the component surface due to the relative motion between the fiber nozzle and the component.

Due to the thermal capacity of the roving and the bending stiffness of the roving, the roving dives into the liquid zone in the component. A coating hinders the roving from buckling or jamming, thus preventing the fiber nozzle in the fiber integration unit from clogging. The melted polymer wets the supplied roving and fastens it in the component’s material. The cutting blade is then used to cut the roving to a length programmed in the NC code. The residual length of roving remaining in the roving extruder after cutting is pulled out of the fiber nozzle to the end of the roving path. Following the successful integration of all rovings within the designated layer, the fiber integration unit proceeds to its zero-point. Thereafter, fresh powder is applied. After the IR emitters have heated the applied powder surface to the sintering temperature, the laser melts the applied powder layer locally. At this moment, the roving is completely embedded in the polymer matrix. This process is repeated iteratively until all rovings have been integrated or the component has been completed. Finally, the machine is cooled in a controlled manner [6].

### 4.3. Properties of CoFRP by Laser Sintering and Evaluation

Parts produced by this process have been characterized with regard to FVC and their respective tensile properties. A maximum FVC of 25.1% could be achieved with this new process. For this FVC, a Young’s modulus of 52 GPa with a tensile strength of 417 MPa was measured. The elongation at break was determined to be 0.87%. Compared to unreinforced PA12, this is an increase in stiffness of 3050%, in strength of 800%, and in elongation at break of approximately −100% [50].

In consideration of the fracture behavior, fiber pullout was predominantly observed, attributable to the inadequate impregnation of the roving with the matrix. The selection of the applied acrylic coating (PERICOAT AC 250) was not driven by its matrix compatibility but rather by the enhanced feedability of the roving, which resulted in an increased stiffness. The application of the coating was restricted to the outer periphery of the roving, with the core remaining uncoated and dry. This approach resulted in suboptimal load transfer into the fibers, as the core of the roving functioned as an extensive pore, thereby creating a weak point within the composite structure. Consequently, the resulting stiffness and strength values of the composite fell below the anticipated levels.

## 5. Comparisons

In the literature, different polymers have been used compared to the ones investigated in this research. The reason for this was the focus on technical relevance regarding the process. For instance, the primary strength of APF lies in its employment of injection molding granulates for three-dimensional printing applications. This capability enables the utilization of certified materials for prototyping, which are typically employed in serial production. To facilitate a comparative analysis of composites with diverse matrix and fiber materials, the fulfillment factor is employed. This factor is calculated by determining the percentage of material properties that have been experimentally attained in comparison to the theoretical maximum, as predicted by the Voigt model [51]. $D_{F,E}$ and $D_{{F,R}_{m}}$ denote the degree of fulfilment regarding Young’s modulus (*E*) and maximum strength (*R_m_*), respectively. They result from the ratio of the experimental (*exp*) values compared to the theoretical values (*Voigt*) from Voigt’s model.

(1) $$D_{F,E}=\frac{E_{exp}}{E_{Voigt}}$$

(2) $$D_{{F,R}_{m}}=\frac{R_{m,exp}}{R_{m,Voigt}}$$

with the respective theoretical values of

(3) $$E_{Voigt}=\varphi *E_{F}+\left(1-\varphi \right)*E_{M}$$

(4) $$R_{Voigt}=\varphi *R_{F}+\left(1-\varphi \right)*R_{M}$$

where *φ* represents the FVC. The indices of *F* and *M* denote fiber and matrix values, respectively. Table 1 lists the fulfilment factors of the previously introduced processes. The new processes presented in this study can be found in the bottom rows and are marked accordingly. In order to have further comparative values, the table also contains values for the FFF process.

The elevated mean fulfillment factor for Young’s modulus across all processes can be attributed to the inherent characteristics of reinforcing fibers. The length of fibers directly correlates with their effectiveness in enhancing stiffness, with shorter lengths resulting in greater impact strength [52]. In the context of additive manufacturing, the process-induced porosity and the suboptimal bonding between fibers and the matrix can reduce the effective fiber length, thereby leading to a stiffening effect more pronounced than the effect on strength.

Significant variability is observed in the FFF processes, with the fulfillment factor ranging from 10% to 77% for stiffness (average: 52%) and from 14% to 84% for strength (average: 37%). The presence of a lack of a separate consolidation process leads to a high pore content between the fibers and the thermoplastic, resulting in sometimes very low fulfillment factors and otherwise high differences.

APF demonstrates significantly superior fulfillment factors for stiffness (average: 95%) but comparatively lower performance in strength (average: 23%) in comparison to the FFF process. This disparity can be attributed to the absence of a distinct consolidation process, which results in a high content of pores between the fibers and the thermoplastic. Additionally, the decline in strength fulfillment with higher FVC can be attributed to the interactions between the placed fibers and the generation of additional flaws in the composite.

The novel DES process, when utilized in conjunction with tape laying, exhibited an enhancement in the fulfillment factor, attributable to the supplementary consolidation steps that culminated in values that surpassed the mean for strength (67%) when benchmarked against alternative processes. However, it is imperative to note that the process has been evaluated solely with a single material system to date, necessitating further experimentation to ascertain its general applicability. Nonetheless, the efficacy of the consolidation step is likely the primary factor contributing to the observed enhancement in strength.

Finally, CoFRP, produced by the novel LS process, exhibited above-average stiffness (85%) and average strength (39%). This outcome is attributed to suboptimal process parameters and defects in the compound. Insufficient fiber–matrix bonding and interactions between neighboring fibers significantly influence the resulting properties.

For all three of the newly developed processes, the underlying reasons, specifically the interactions between the process and the resulting material properties, require further investigation.

## 6. Conclusions

The application of FFF and APF processes seems to be limited to prototyping or low-load applications without later consolidation. For FFF processes, the low investment cost and the fast production face the necessity of pre-processed material in the form of filaments. Conversely, APF offers the possibility of employing industrial-grade polymer granulates, yet it is encumbered by high investment costs. Both processes are limited in their build volume. DES, on the other hand, offers a larger build volume but comes with high investment costs and typically higher tolerances. The possibility for the use of granulates and the out-of-plane reinforcement poses an intriguing alternative. The dedicated consolidation process also generally ensures better fiber–matrix bonding and, thus, a higher degree of fulfillment. Lastly, the LS process is best suited for the production of smaller batches and near-finished parts, due to the higher productivity and end-use ready surface quality. The necessary post-processing steps (removal of excess powder) are automated by now.

## Figures and Tables

**Table 1:** Fulfilment factors of selected CoFRP produced using different processes.

Process	Source	Material	FVC of co-Fibers [%]	$D_{F,E}$ [%]	$D_{F,R_{m}}$
MEX (FFF)	[33]	CF, PA6	15	18.9	44.3
	[34]	GF, Nylon	8	49.3	47.5
	[34]	GF, Nylon	10	48.2	53.7
	[34]	CF, Nylon	11	23.1	41.8
	[35]	GF, Nylon	35	27.8	36.6
	[35]	GF, Nylon	41	10.5	34.1
	[36]	GF, Nylon	50	70.4	33.2
	[36]	CF, Nylon	40	56.5	40.8
	[37]	CF, Nylon	27	76.8	83.7
	[13]	GF, Nylon	25	26.9	16.8
	[13]	GF, Nylon	60	20.2	13.8
	[38]	CF, Nylon	35	44.2	37.1
**APF**	[39]	**GF, ABS**	**15**	**94.7**	**27.4**
	[39]	**GF, ABS**	**18**	**93.5**	**22.3**
	[39]	**GF, ABS**	**21**	**94.9**	**18.5**
**DES**	[15]	**CF, PA6CF30**	**3**	**not measured**	**67.4**
**LS**	[6]	**CF, PA12**	**25**	**84.6**	**38.5**

## Data Availability

Data are contained within the article.
